# Histone modifications as markers of cancer prognosis: a cellular view

**DOI:** 10.1038/sj.bjc.6603844

**Published:** 2007-06-26

**Authors:** S K Kurdistani

**Affiliations:** 1 Department of Biological Chemistry, David Geffen School of Medicine, University of California, Los Angeles, CA 90095-1737, USA

**Keywords:** epigenetics, histone modifications, prognosis, biomarker

## Abstract

Alterations in modifications of histones have been linked to deregulated expression of many genes with important roles in cancer development and progression. The effects of these alterations have so far been interpreted from a promoter-specific viewpoint, focussing on gene–gene differences in patterns of histone modifications. However, recent findings suggest that cancer tissues also display cell–cell differences in total amount of specific histone modifications. This novel cellular epigenetic heterogeneity is related to clinical outcome of cancer patients and may serve as a valuable marker of prognosis.

Epigenetics is the study of the inheritance of phenotypes that occur without a corresponding change in DNA sequence. The molecular mechanisms of epigenetic inheritance as it relates to chromatin include the three inter-related processes of DNA methylation, genomic imprinting and histone modifications (Kouzarides, 2007). Through small chemical moieties that covalently attach to DNA or histones, the epigenetic processes can increase the capacity of the genome to store and transmit biological information beyond the DNA sequence. In cancer, in addition to sequence alterations in the genome, there are also changes in the epigenetic information (Jones and Baylin, 2002; Hake *et al*, 2004). Both DNA methylation, including genomic imprinting, and histone modifications show altered patterns of distribution in cancer cells. The epigenetic alterations may occur at different stages of tumourigenesis and thus contribute to the development and/or progression of cancer. While different cancers share certain phenotypic hallmarks such as unregulated growth or resistance to apoptosis (Hanahan and Weinberg, 2000), there is a great degree of heterogeneity in both genetic and epigenetic alterations that may give rise to these similar phenotypes. Such genetic and epigenetic heterogeneity may underlie the varied clinical behaviour of cancers that can range from indolent, slow-growing to aggressive, fast-growing tumours. A challenge in cancer therapy is to be able to predict the behaviour of the cancer at an early stage so that appropriate treatments are administered to patients (Ludwig and Weinstein, 2005). Clinical outcome prediction is based generally on grade, the degree of tumour differentiation and/or stage, a measure of tumour size and spread beyond the primary site. However, within cancers that are of equivalent grade and stage, there are subtypes of patients that are molecularly heterogeneous and have different clinical outcomes. In this regard, molecular biomarkers such as gene expression have been useful in distinguishing subtypes of cancer patients with distinct clinical outcomes, thereby expanding our prognostic capabilities.

Similar to gene expression, epigenetic factors such as DNA methylation of specific genes have also been used as biomarkers but to a much lesser extent. In fact, despite a long and growing list of genes with altered DNA methylation in cancer, only a few have been related to clinical outcome of cancer patients. Even less is known about the association between histone modifications and outcome of cancers. While no promoter-specific histone modifications have been related to cancer prognosis, recent work indicates that patterns of certain histone modifications, not at specific genes, but at the level of individual cells, can be used to predict cancer outcome (Seligson *et al*, 2005). In this review, I will briefly highlight the current ‘epigenetic prognosticators’ with emphasis on histone modifications, explore the potential underlying mechanisms and how they may be used to increase the effectiveness of epigenetic drugs such as histone deacetylase inhibitors (HDACi).

## DNA METHYLATION IN CANCER

Following the seminal discovery that normal and cancer cells show differential methylation patterns, cancer epigenetics has been by and large focussed on DNA methylation (Feinberg and Tycko, 2004). Over two decades of research has shown that, compared to normal cells, DNA of cancer cells is generally hypomethylated, while promoters of certain genes are hypermethylated, most commonly in the context of CpG islands. Such promoter-specific increase in methylation leads to silencing of the affected gene that might have functioned as, for instance, a tumour suppressor. Transcriptional repression by DNA methylation is mediated by a class of methyl DNA binding proteins which, by virtue of recognising specifically methylated DNA sequences, recruit repressive protein complexes including histone deacetylases (HDACs) to gene promoters (Klose and Bird, 2006). The combination of CpG island methylation, proteins that binds to them, and repressive histone modifications generates localised regions of specialised chromatin, which can inhibit transcription.

Despite a growing list of genes including tumour suppressors and DNA repair genes that are aberrantly hypermethylated in different cancers, only a limited number of the identified hypermethylated genes have demonstrated any potential utility in clinical decision making. Nonetheless, in recent years, evidence for clinico-pathological significance of gene-specific DNA methylation has been emerging. In gliomas, methylation of the O^6^-methylguanine-DNA methyltransferase (MGMT) gene promoter is a predictor of patients' responsiveness to alkylating agents (Esteller *et al*, 2000). O^6^-methylguanine-DNA methyltransferase is a DNA repair protein that prevents formation of toxic DNA inter-strand crosslinks. When MGMT promoter is methylated, the gene is not transcribed, leading to enhanced sensitivity of cells to alkylating agents. Moreover, the predictive power of promoter methylation may be increased when multiple promoters are examined at once. In neuroblastoma, unbiased clustering of several methylated CpG islands has been linked to clinical outcome (Alaminos *et al*, 2004). As opposed to single-gene analysis, the integrated information on methylation patterns of multiple genes may reflect the functional status of several cellular pathways and prove to be a reliable predictor of prognosis.

Loss of imprinting (LOI) – loss of parent-of-origin DNA methylation pattern – may also contribute to carcinogenesis. For instance, LOI of the IGF2 (insulin-like growth factor 2) gene is seen in many patients with colorectal cancer, and biallelic expression of IGF2 is found in the normal-appearing colonic epithelium of colorectal cancer patients. Elevated levels of IGF2 in normal cells may confer a growth advantage that is necessary for tumour initiation (Sakatani *et al*, 2005).

## HISTONE MODIFICATIONS IN CANCER BIOLOGY

In addition to DNA methylation, modifications of histones are important determinants of epigenetic state. In eukaryotes, an octamer of histones H2A, H2B, H3 and H4 is wrapped by 147 bp of DNA to form a nucleosome – the fundamental unit of chromatin (Luger *et al*, 1997). Histones are subject to a variety of post-translational modifications, especially on their N termini, including acetylation (ac) of lysines (K) and methylation (me) of lysines and arginines (R) as well as phosphorylation, ubiquitylation, glycosylation, sumoylation, ADP-ribosylation and carbonylation. These modifications play fundamental roles in gene regulation and other chromatin-based processes. The enzymes that modify histones show distinct specificities and distinguish between histone subtypes as well as individual residues within a given histone (Suka *et al*, 2001). The histone-modifying enzymes affect histones either locally, through targeted recruitment by sequence-specific transcription factors (Rundlett *et al*, 1998), or globally throughout the genome in an untargeted manner affecting virtually all nucleosomes (Vogelauer *et al*, 2000). Such widespread functions that occur independently of apparent sequence-specific DNA-binding proteins are referred to as ‘global’ histone modifications. Like their targeted effects, the global activity of the histone-modifying enzymes can modulate gene activity (Vogelauer *et al*, 2000). Therefore, histones are modified locally and globally through multiple histone-modifying enzymes with different substrate specificities, generating hierarchical patterns of modifications from single promoters to large regions of chromosomes and even single cells. For instance, heterochromatin – highly repetitive, transcriptionally inactive and late-replicating regions of the genome – is generally deacetylated but could be enriched for H3K9me2/3 (Horn and Peterson, 2006). This is in contrast to euchromatin – gene rich and transcriptionally active regions of the genome – which is associated with H3K4me2 and increased acetylation (Strahl *et al*, 1999). Therefore, genomic distribution of specific histone modifications may reflect the underlying organization of the genome.

Considering the fundamental roles of histone modifications, it is not surprising that aberrations in histone modifications are discovered in cancer. Essentially all these aberrations have been shown to occur at individual gene promoters due to inappropriate targeting of one or more histone-modifying enzymes. For instance, the E2F transcription factor recruits the tumour suppressor retinoblastoma protein (Rb) to its target genes. Rb in turn recruits HDAC1 that leads to transcriptional silencing of genes with important roles in tumour biology such as cyclin E (Brehm *et al*, 1998). Cancer-related perturbations of promoter-specific epigenetic patterns result in improper repression or activation of genes that could ultimately lead to cellular transformation, carcinogenesis or cancer progression. For instance, chromatin immunoprecipitation (ChIP; a method for determining sites of protein–DNA interactions *in vivo*) experiments show that silencing of the CDKN2A locus – which encodes the p16INK4A and p14ARF tumour suppressors – is associated with hypermethylation of H3K9 and hypomethylation of H3K4 (Nguyen *et al*, 2002). Aberrant modification of histones associated with DNA repetitive sequences has also been reported. These aberrations include lower levels of histone H4K16ac and K20me3 in haematological malignancies and colorectal adenocarcinomas (Fraga *et al*, 2005).

## HISTONE MODIFICATIONS IN CANCER PROGNOSIS

While cancers have atypical patterns of histone modifications, none of the locus-specific changes in histone modifications has so far been related correlatively or causally to clinical outcome. This is perhaps because deregulation of histone modifications at the level of a single promoter is intimately linked to mis-expression of the downstream gene, which in most cases may not provide adequate information to predict clinical outcome.

In contrast to promoter-targeted histone modifications, we have shown recently that cellular levels of histone modifications are related to cancer prognosis (Seligson *et al*, 2005). Our initial interest in the roles of histone modifications in cancer was derived from our findings in yeast. Mapping patterns of histone modifications at promoters to gene expression in yeast suggested that histones can store biological information in their modification patterns (Kurdistani *et al*, 2004). Extending this idea to cancer, we wondered whether promoter-specific histone modification patterns, on a genomewide scale, encode information that can be used to predict prognosis. Determining gene-specific patterns of histone modifications of cancer cells in primary tissue specimens can be cumbersome due to cellular heterogeneity and tissue quantity, and would involve high-throughput ChIP which may not be easily adaptable by clinical laboratories. Thus, we turned our attention to cellular patterns of histone modifications.

**Figure 1:**
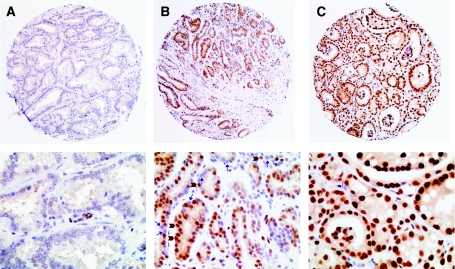
Cellular epigenetic heterogeneity in cancer. Immunohistochemical examination of prostate cancer tissues by an antibody against histone H3 lysine 18 acetylation reveals cell–cell differences in total levels of H3K18ac. The cells with brown nuclei are positively stained (indicated by brown block arrows) and their increased percentage is related to better clinical outcome. The unstained nuclei are blue (indicated by blue arrows). The tissues shown are of equivalent grade but represent (**A**) low, (**B**) medium and (**C**) high levels of cellular H3K18ac. Magnification: 10 × , top panel; 40 × , bottom panel.

Since histone modifications occur throughout the genome, any potential change in the activity or specificity of the histone-modifying enzymes should result in detectable changes in specific histone modifications at the level of individual nuclei by immunostaining. This is indeed the case. Immunohistochemical (IHC) examination of primary prostate cancer tissues with antibodies against specific histone modifications revealed that cancer cells display much heterogeneity in both the fraction of cells that are positively stained and the intensity of staining (Figure 1), parameters that are scored routinely by pathologists. Such heterogeneous cellular staining was evident for all histone modifications examined which included H3K4me2, K9ac, K18ac, H4R3me2 and K12ac (Seligson *et al*, 2005). While the IHC approach provides no information on the genomic, gene–gene differences in distribution of histone modifications, it reveals novel cell–cell heterogeneity in histone modification levels that would be hidden in molecular approaches such as ChIP.

Is the cellular heterogeneity in histone modification levels related to prostate cancer outcome? Analysis of the levels of each modification individually was not informative of prognosis, which was defined as time to tumour recurrence after surgical removal of the primary tumour. When the data from all modifications were analysed collectively, the histone modification patterns defined two groups of patients with significantly different risks of tumour recurrence, independently of other clinico-pathological features. A second prostate cancer TMA validated the prognostic power of two of the histone modifications, H3K4me2 and K18ac (Seligson *et al*, 2005). So, the histone modification patterns provide additional, non-redundant prognostic information to the known prognostic features of prostate cancer. This finding suggests for the first time that epigenetic heterogeneity between cancer tissues may underlie the varied outcome of the disease. Surprisingly, the patient group with lower cellular levels of histone modifications (i.e. decreased percent cell staining) had poorer prognosis. This seems counterintuitive as the examined histone modifications are all associated with gene activity, and so, their loss should have adverse effects on gene expression and tumour growth. However, lower cellular levels by IHC may represent, at a molecular level, re-distribution of a histone modification from most histones throughout the genome to a limited number of genes. These genes may then confer a more aggressive phenotype to the tumour. Alternatively, reduction of global histone modification levels may reflect conversion of chromatin from a euchromatic to a heterochromatic state. Such ‘heterochromatinization’ of the genome may provide cells a protective measure against genotoxic stress by limiting DNA exposure. Although these scenarios are mere hypotheses, they can be tested by molecular examination of histone modifications in tumours with different cellular levels. Interestingly, a recent study provides some evidence that cellular levels of histone modifications in prostate differ between benign, pre-neoplastic and cancer tissues, suggesting that histone modification patterns may be dynamic and change during various steps of carcinogenesis (Mohamed *et al*, 2007).

The aberrations in cellular levels of histone modifications are consistent with findings that histone-modifying enzymes have impaired activity in various cancers (Esteller, 2006). For instance, missense and truncating mutations of p300 histone acetyltransferase (HAT) and loss of heterozygosity at the p300 locus, which could result in global reduction in histone acetylation, are associated with multiple cancers (Gayther *et al*, 2000). In leukaemia, p300 is fused to MLL through a translocation (Ida *et al*, 1997), which may confine its HAT activity only to MLL-target genes and result in diminished histone acetylation from most of the genome. Since the balance of HAT and HDAC activities determines the global levels of histone acetylation, impaired HDAC activity could also contribute to cellular patterns of histone acetylation. In fact, HDACs show deregulated expression in multiple cancers (Ozdag *et al*, 2006), and HDAC6 expression may be related to clinical outcome in breast cancer (Zhang *et al*, 2004; Krusche *et al*, 2005). A truncating mutation abolishes HDAC2 enzymatic activity in several sporadic tumours (Ropero *et al*, 2006). Histone methyltransferases (HMTs) are also deregulated in cancer and may affect global methylation levels. In prostate cancer, lysine-specific demethylase 1 (LSD1) overexpression is associated with increasing grade and may be used to predict prognosis (Kahl *et al*, 2006). The polycomb group protein enhancer of zeste homologue 2 (Ezh2) is a H3K27 HMT which is required for cell proliferation, is over-expressed in metastatic prostate cancer and is associated with poor prognosis (Varambally *et al*, 2002). The activity of histone-modifying enzymes can also be regulated through post-translational modifications of the enzyme complexes themselves. For instance, the kinase Akt phosphorylates Ezh2, suppressing Ezh2 HMT activity by reducing its affinity for histone H3, leading to global loss of histone methylation at K27 (Cha *et al*, 2005). Finally, histone-modifying enzymes commonly reside in multi-protein complexes. Changes in expression of one or more component of these complexes may not only affect the activity of the complex but also lead to formation of complexes with altered histone substrate specificities. For instance, overexpression of Ezh2 in tissue culture promotes formation of a new polycomb repressive complex 4 that acquires new substrate specificity, methylating linker histone H1 in addition to H3K27 (Kuzmichev *et al*, 2005). So, while mis-targeting of histone-modifying enzymes can generate aberrant local, promoter-specific patterns of histone modifications, the altered activity of the enzyme complexes, by mutations, expression changes, or post-translational control, may provide a mechanism to regulate histone modifications throughout the genome.

## CELLULAR HISTONE MODIFICATION PATTERNS AND HDAC THERAPY

The prognostic cellular histone modification patterns may not only be used as general biomarkers but could have specific implications for therapies involving inhibitors of HDAC (HDACi) or other enzymes such as histone demethylases. The patients with poorer outcome who have low levels of H3K4me2 and K18ac could perhaps benefit more from HDACi or require a different regimen of various epigenetic therapeutics compared to those with high levels of both histone modifications. The current generation of HDACi is mostly not selective and can inhibit multiple HDACs (Minucci and Pelicci, 2006). Given the distinct *in vivo* specificities of histone-modifying enzymes, IHC analysis of histone modifications may also reveal which specific modifications show the most reduction, guiding the design and use of drugs with more restricted targets.

**Figure 2:**
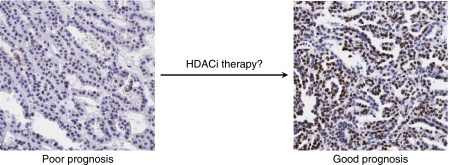
A hypothetical effect of HDAC inhibitors on cellular epigenetic patterns in cancer. In addition to their promoter-specific effects, inhibitors of histone deacetylases (HDACis) may also increase the prevalence of cells with high acetylation levels of specific histone residues. This would shift the cellular epigenetic patterns from a poor (left) to better (right) prognosis. Histone demethylase inhibitors may have a similar effect on cellular methylation patterns.

Histone deacetylase inhibitors have pleiotropic effects on cancer cells including growth arrest, apoptosis and differentiation. These effects are linked to transcriptional reactivation of certain genes such as p21, which causes growth arrest (Richon *et al*, 2000). However, the cellular heterogeneity in histone modification patterns suggests the possibility that HDACi may also have broader effects. Histone deacetylase inhibitors may reduce the heterogeneity in cellular levels of histone modifications by increasing the prevalence of cells with higher levels of histone acetylation. This would shift the cellular patterns to the more favourable prognostic category (Figure 2). Since lower cellular histone methylation levels are also associated with poorer prognosis, demethylase inhibitors, perhaps in combination with HDACi, could have additive or synergistic effects on modifying the cellular patterns (Metzger *et al*, 2006).

## CONCLUSION

Cancer is widely considered to have a clonal origin but cancer tissues are comprised of a heterogeneous group of cells with different functional potential. Analysis of histone modifications of individual cells in primary cancer tissues has now revealed a new layer of heterogeneity: cell–cell differences in total amount of acetylation and methylation of specific histone residues. This cellular epigenetic heterogeneity is related to the clinical outcome of cancer patients. While promoter-specific histone modification patterns can be established through targeted recruitment of histone modifiers, much work remains to elucidate the molecular determinants of variation in cellular levels of histone modifications. An ‘epigenetic model of cancer’ has been proposed to account for epigenetic contribution to cancer development and progression (Baylin and Ohm, 2006; Feinberg *et al*, 2006). In this model, promoter-specific epigenetic alterations can result in mis-regulation of genes which increase the risk of cancer development and contribute to cancer progression. Whether and how the cellular patterns of histone modifications can be incorporated into this model remains an exciting endeavour.
